# Prognostic factors in the acute respiratory distress syndrome

**DOI:** 10.1186/s40169-015-0065-2

**Published:** 2015-07-02

**Authors:** Wei Chen, Lorraine B Ware

**Affiliations:** Division of Allergy, Pulmonary, and Critical Care Medicine, Department of Medicine, Vanderbilt University School of Medicine, Nashville, USA; Division of Pulmonary and Critical Care Medicine, Chiayi Christian Hospital, 600 Chia-Yi, Taiwan; Department of Life Science, National Chung Hsing University, Taichung, Taiwan; Department of Respiratory Therapy, China Medical University, Taichung, Taiwan; Department of Pathology, Microbiology and Immunology, Vanderbilt University School of Medicine, Nashville, USA

**Keywords:** Acute respiratory distress syndrome, Prognostic factors, Biomarkers, Oxygenation, Scoring system

## Abstract

Despite improvements in critical care, acute respiratory distress syndrome (ARDS) remains a devastating clinical problem with high rates of morbidity and mortality. A better understanding of the prognostic factors associated with ARDS is crucial for facilitating risk stratification and developing new therapeutic interventions that aim to improve clinical outcomes. In this article, we present an up-to-date summary of factors that predict mortality in ARDS in four categories: (1) clinical characteristics; (2) physiological parameters and oxygenation; (3) genetic polymorphisms and biomarkers; and (4) scoring systems. In addition, we discuss how a better understanding of clinical and basic pathogenic mechanisms can help to inform prognostication, decision-making, risk stratification, treatment selection, and improve study design for clinical trials.

## Introduction

The acute respiratory distress syndrome (ARDS) is a devastating syndrome of pulmonary inflammation and edema manifested clinically by the acute onset of bilateral infiltrates on chest radiograph, and arterial hypoxemia [1–3]. Despite advances in the care of critically ill patients, both short and long-term mortality rates of ARDS remain high [4]. Currently, the only therapy that has been definitively proven to reduce mortality in ARDS is a lower tidal volume ventilator strategy [5]. However, other therapies have been shown to reduce mortality in a subset of patients with ARDS. For example, application of prone positioning in patients with severe ARDS (defined for this study as a ratio of the partial pressure of arterial oxygen to the fraction of inspired oxygen [PaO2/FiO2] of less than 150 mm Hg) significantly decreased 28-day and 90-day mortality [6]. Thus, a better understanding of prognostic factors in ARDS is crucial for facilitating risk stratification and developing new therapeutic interventions that aim to improve clinical outcomes. The aim of this article is to review known clinical predictors and biologic markers of mortality in ARDS. We categorize the prognostic factors into four areas including (1) clinical characteristics; (2) physiological parameters and oxygenation; (3) genetic polymorphisms and biomarkers; and (4) scoring systems. In addition, we discuss how a better understanding of clinical and basic pathogenic mechanisms can help to inform prognostication, decision-making, risk stratification, treatment selection, and improve study design for clinical trials.

## Review

### Clinical characteristics

There have been a number of large studies over the past several decades that have explored clinical risk factors for hospital and short-term mortality in ARDS. Clinical predictors that were consistent across multiple studies include older age, worse physiologic severity of illness (as measured by severity scores such as the Acute Physiology and Chronic Health Evaluation (APACHE) or Simplified Acute Physiology Score (SAPS)), shock on hospital admission, arterial pH less than 7.30, liver disease, early air leak, immunosuppression, triggering risk factor, and right ventricular dysfunction [7–11]. It is important to note that these studies were all done before the era of lower tidal volume ventilation. In more recent studies done in clinical trial patient populations after the widespread implementation of low tidal volume ventilation, severity of illness as measured by APACHE II or APACHE III has remained a robust clinical predictor of mortality as have age, and the presence of non-pulmonary organ failures [12, 13].

In regards to effects of race and ethnicity on mortality from ARDS, a retrospective cohort study from the National Institutes of Health (NIH) ARDS Network revealed that African American and Hispanic patients have a higher mortality from ARDS than Caucasian patients [14]. The mechanisms that underlie this racial disparity in mortality are unknown; one proposed factor is related to the high incidence of a mutation in the promoter region of the Duffy antigen/receptor for chemokines (Darc) gene in those of African ancestry that leads to low levels of erythrocyte Duffy antigen, reduced chemokine binding and potentially to higher levels of circulating chemokines such as IL-8 [15]. In a large multicenter study, Lemos-Filho LB et al. found that African-American patients were less likely to develop ARDS than Caucasians even after adjustment for clinical predictors despite having higher severity of illness at presentation. In the same study, there was no difference in ARDS mortality by sex or race after adjustment for covariates [16]. Recently, Reilly et al. reported that blood type A is a risk factor for ARDS in Caucasian patients with major trauma and sepsis [17]. However, it is not known whether the same blood type is also associated with poor prognosis in ARDS and this study has yet to be validated in another patient population.

The role of diabetes in the development of ARDS is still controversial. Koh et al. showed that diabetes mellitus is not associated with development of ARDS in a large cohort of critically ill patients [18], although patients with diabetes had higher mortality regardless of ARDS status. By contrast Yu et al. reported a protective effect of diabetes on development of ARDS [19], but diabetes had no association with outcomes.

Most of the original studies of clinical risk factors for mortality in ARDS have focused on short-term outcomes such as hospital or 28-day mortality. However, it is increasingly clear that long-term mortality after ARDS and other critical illness is substantially higher than short-term mortality; long-term mortality may be driven by different factors than short-term. A multi-intensive care unit, prospective cohort study of 646 patients hospitalized with ARDS showed that mortality at 1 year was substantially higher than in-hospital mortality (41 % vs. 24 %, *p* < 0.001). The independent predictors of death at 1 year were age, living somewhere other than home prior to admission, and serious comorbidities such as HIV and malignancy [20]. For very severe ARDS patients requiring extracorporeal membrane oxygenation (ECMO) intervention, Schmidt et al. constructed a mortality prediction model with eight pre-ECMO parameters, i.e., age, body mass index, immunocompromised status, prone positioning, days of mechanical ventilation, sepsis-related organ failure assessment, plateau pressure and positive end-expiratory pressure; this scoring system accurately predicted long term mortality in this select group of severely ill ARDS patients [21].

### Physiological parameters and oxygenation

#### Physiological parameters

A variety of physiological parameters are associated with adverse outcomes in patients with ARDS. In the landmark study of lower tidal volume ventilation for ARDS from the NIH ARDS Clinical Trials Network, inspiratory plateau pressures were significantly lower on days 1, 3, and 7 in the group treated with lower tidal volumes with lower mortality compared to the group treated with higher tidal volumes with higher mortality [5]. In the ALVEOLI study of two different levels of positive end expiratory pressure in ARDS, the alveolar-arterial oxygen gradient and inspiratory plateau pressure were good predictors for mortality in ARDS patients [22]. Furthermore, Nuckton et al. found that pulmonary dead-space fraction, which was calculated by the mean expired carbon dioxide fraction and was measured with a bedside metabolic monitor, was an independent risk factor for death [23]. Several subsequent studies have also reported that an elevated ratio of pulmonary dead space to tidal volume (VD /VT) within 24 h of onset of ARDS was associated with higher mortality [24, 25]. Elevated pulmonary dead space fraction likely reflects the extent of pulmonary microvascular thrombosis and injury or frank destruction of the pulmonary vascular bed, providing an important index of the severity of acute lung injury.

Beyond respiratory parameters, there is controversial data regarding the effect of right heart failure on the prognosis of ARDS. In a large multicenter cohort study in Europe, a higher ratio of right over left ventricular stroke work (RVSW/LVSW) at admission was independently associated with mortality [26]. However, in a randomized control study with a total of 145 ARDS patients receiving pulmonary artery catheters (PAC), there was no difference in 90-day mortality between patients with and without right ventricular failure (71 vs. 67 %, respectively) [27]. Again, it should be noted that the first study was done before the era of low tidal volume ventilation; ventilation with higher tidal volumes and higher pulmonary distending pressures may have had adverse effects on right heart function that were not as apparent in the more recent study. In another study in the low tidal volume ventilation era, Bull et al. demonstrated that pulmonary vascular dysfunction, which they defined as elevated transpulmonary gradient and pulmonary vascular resistance index measured by PAC in patients enrolled in the NIH ARDS Network Fluid and Catheter Treatment Trial [28], was an independent risk factor for 60-day mortality [29].

#### Oxygenation

It seems intuitive that the severity of hypoxemia should be associated with adverse outcomes in ARDS. However, over the past two decades, several studies have shown that the PaO_2_/FiO_2_ measured at the onset of ARDS is not an independent predictor of mortality [9, 30–34]. One possible explanation for the inability of initial PaO_2_/FiO_2_ to discriminate adverse outcomes in ARDS is that the PaO_2_/FiO_2_ varies depending on ventilator strategy. Indeed, manipulation of ventilator settings including tidal volume (Vt) and positive end expiratory pressure (PEEP) can greatly influence the values of PaO_2_/FiO_2_ [35]. Since PaO_2_/FiO_2_ is so sensitive to ventilator settings, some investigators have asked whether the prognostic value of PaO_2_/FiO_2_ might improve with standardized ventilator settings. In a prospective, multicenter study of 452 ARDS patients with PaO_2_/FiO_2_ measured on standard ventilator settings of volume assist/control mode, tidal volume 7 ml/kg PBW, inspiratory: expiratory time ratio (I:E) <1:1, ventilator rate to maintain PaCO_2_ of 35–50 mmHg and FiO_2_ and PEEP settings applied in the following order: (1) FiO_2_ ≥ 0.5 with PEEP ≥ 5 cmH2O, (2) FiO_2_ ≥ 0.5 with PEEP ≥ 10 cmH2O, (3) FiO_2_ = 1.0 with PEEP ≥ 5 cmH2O, and (4) FiO_2_ = 1.0 with PEEP ≥ 10 cmH2O, risk stratification of ARDS was substantially improved. Of these settings, only the PaO_2_/FiO_2_ ratio measured using PEEP ≥ 10 cmH2O and FiO2 ≥ 0.5 at 24 h after ARDS onset showed substantial improvement in risk stratification for short term ARDS mortality [36]. The finding is also consistent with analyses published with the Berlin ARDS definition; mortality prediction in ARDS was better when based not only on PaO_2_/FiO_2_, but also on PEEP level and the extent of lung infiltrates in chest radiograph [1]. Bone et al. showed that early improvement of PaO_2_/FiO_2_ with conventional therapy is a prognostic factor of better outcome in patients with ARDS [30]. In addition, the oxygenation response to the titration of PEEP or other ventilator settings, such as high-frequency oscillatory ventilation, may also be prognostically useful in ARDS [37, 38]. Based on these findings, clinical trials that use the PaO_2_/FiO_2_ to select patients with more severe ARDS for enrollment have become more prevalent. A recent multicenter, prospective, randomized, controlled trial of prone positioning in patients with severe ARDS as defined by PaO_2_/FiO_2_ < 150 mmHg on PEEP of ≥ 5 showed that prolonged prone-positioning sessions significantly decreased mortality in this patient group [6].

Unlike adults, children with ARDS are less likely to have complicated underlying diseases or other organ involvement. Thus, Flori et al. showed that the initial severity of arterial hypoxemia correlated well with ICU mortality in 328 children with ARDS [39]. Furthermore, Wong et al. showed that both a low PaO_2_/FiO_2_ ratio and a low SpO_2_/FiO_2_ were associated with poor outcomes in children with ARDS [40].

The Oxygenation Index (OI) is defined as the reciprocal of PaO_2_/FiO_2_ ratio multiplied by the mean airway pressure and was originally developed for evaluation of candidates for extracorporeal membrane oxygenation in pediatric respiratory failure [41]. The OI has also been evaluated as a clinical predictor in ARDS. In a study of 149 patients with ARDS undergoing lung protective ventilation, OI was superior to PaO_2_/FiO_2_ ratio, and was an independent predictor of mortality [42]. Similarly, a high OI at 24 h of ARDS was a risk factor for mortality [40], and improvement in OI during the first 24 h predicted a better outcome in pediatric patients with ARDS [43].

### Genetic polymorphisms and biomarkers

#### Polymorphisms

Several factors have made it challenging to determine the influence of genetic heterogeneity on clinical outcomes in ARDS. Foremost is the fact that ARDS is a complex clinical syndrome that involves multiple pathogenetic pathways and affects a diverse spectrum of patients who often have comorbid illnesses [3]. Additionally, it has been difficult to accrue sufficient patient numbers for identification and validation of genetic risk modifiers with relatively modest effect sizes. Nevertheless, a variety of genetic polymorphisms related to inflammation, innate immunity, epithelial cell function, and angiogenesis have been reported to be associated with adverse outcomes of ARDS [15, 44–48] as shown in Table 1. Although a variety of common polymorphisms are associated with adverse outcomes in ARDS, such as homozygosity for the 4G allele of plasminogen activation inhibitor-1 (PAI-1) [49], others such as polymorphisms in the extracellular superoxide dismutase gene have been shown to have an association with better outcomes in ARDS [50]. In addition to the need for larger validation studies to confirm the observed associations, substantial additional research is needed to understand the functional impact of the various candidate polymorphisms on protein function and expression levels and how these functional changes contribute to alterations in ARDS outcomes.

**Table 1:** Polymorphisms associated with mortality in ARDS patients

Gene	No. of patients	Results	Reference
DARC (rs2814778)	132	17 % increase in 60-day mortality	[15]
NFE2L2 (rs6721961)	750	Increased 28-day mortality (OR 9.73)	[45]
NAMPT (rs61330082)	750	Increased 28-day mortality (OR 4.37)	[45]
ADIPOQ (rs208294)	587	Increased mortality (hazard ratio 2.61	[46]
ACE I/D polymorphism, DD type	101	Increase 28-day mortality (OR: 8.8)	[47]
VEGF (−460 T + 405C + 936 T)	394	Increase 28-day mortality (OR: 2.89)	[48]
GCCT haplotype of EC-SOD	157	Decreased 28-day mortality	[50]
PAI-1 4G/5G polymorphism	52	Increase 28-day mortality (OR: 9.95)	[49]

*ACE* angiotensin-converting enzyme, *EC-SOD* extracellular superoxide dismutase, *DARC* duffy antigen/receptor for chemokines, *VEGF* vascular endothelial growth factor

#### Biomarkers

The pathophysiological hallmarks of ARDS are injury to the alveolar epithelial, microvascular endothelial barriers and alveolar-capillary membrane [51], with concomitant activation of inflammatory and coagulation pathways [52]. A variety of protein biomarkers have been studied for their prognostic value in ARDS. For discussion, we have categorized the most promising potential biomarkers into four categories [53], as shown in Table 2 and discussed below.

**Table 2:** Biomarkers associated with mortality in ARDS patients

Categories	Plasma markers	No. of patients	Results	Reference
Endothelium				
	VEGF	40	Elevated VEGF levels in ELF may predict a better outcome	[55]
	VEGF receptor-2	101	Independently predictive of death	[56]
	Angiopoietin-2	931	Independently predictive of death	[59]
	von Willebrand factor	559	Independently predictive of death	[60]
Epithelium				
	Surfactant protein-D	565	Independently predictive of death	[62]
	ELF KL-6	32	Predictive of death	[63]
	RAGE	676	Predictive of death	[65]
	Clara cell protein (CC-16)	78	Predictive of death	[69]
Inflammation				
	LTB4, IL-8	35, 39, 816	Predictive of death	[70–72]
	IL-2, IL-15	34	Predictive of death	[73]
	Decoy receptor (DcR) 3		Predictive of death	[75]
	Ratio of Tregs to all CD4+ lymphocytes in bronchoalveolar lavage (BAL)	47	Predictive of death	[79]
Coagulation				
	Protein C	45, 779	Predictive of death	[83, 84]
	PAI-1	779	Predictive of death	[84]

1. Pulmonary Endothelium

Injury to the pulmonary microvascular bed is a major pathogenetic feature of ARDS. Endothelial injury causes increased vascular permeability primarily at the level of the lung microcirculation, which in turn results in the accumulation of protein-rich pulmonary edema fluid in the alveolus [52]. Thus, various mediators of endothelial activation, injury and permeability have the potential to be prognostic indicators in ARDS.

Vascular endothelial growth factor (VEGF) promotes endothelial survival by inhibiting apoptosis in the alveolar capillary membrane. Abadie et al. reported that VEGF levels in lung tissue obtained by open lung biopsy from 29 ARDS patients were lower when comparing to a control group [54]. Furthermore, ARDS patients with elevated VEGF levels in lung epithelial lining fluid obtained by bronchoalveolar lavage or VEGF-receptor 2 measured in the serum had a better outcome [55, 56].

Angiopoietin-2 (Ang-2) is an angiogenic factor that promotes cell death and vascular destabilization. It is released from endothelial cells in response to endothelial activation by proinflammatory cytokines or local, environmental factors such as hypoxia [57]. Circulating angiopoietin-2 levels are associated with permeability pulmonary edema, and the occurrence and severity of ARDS in patients with and without sepsis [58]. In 931 subjects with ARDS enrolled in a randomized trial of liberal versus conservative fluid management, higher baseline angiopoietin-2 levels were strongly associated with increased mortality in noninfection-related ARDS, but not in infection-related ARDS, indicating that plasma angiopoietin-2 has differential prognostic value for mortality depending on the presence or absence of infection as an ARDS risk factor [59].

Von Willebrand Factor antigen (VWF), a high molecular weight glycoprotein produced predominantly by endothelial cells, can be used as a marker of endothelial activation or injury. In a multicenter study of 559 patients with ARDS, higher plasma levels of VWF were independently associated with adverse outcomes, including mortality, duration of unassisted ventilation, and organ failures [60]. Taken together, the association of lower levels of VEGF and higher levels of Ang-2 and VWF suggests that the extent of endothelial injury is an important determinant of outcomes from ARDS.

2. Pulmonary Epithelium

Surfactant proteins (SP) are secreted primarily by alveolar epithelial type II pneumocytes, and leakage of surfactant proteins from the alveolar space into the circulation is a marker of lung epithelial injury [61]. Eisner et al. showed that baseline SP-D plasma levels were associated with increased risk of death in 565 patients with ARDS. In addition, a low tidal volume protective ventilatory strategy (6 ml/kg) had no effect on the rise in plasma SP-A levels but attenuated the rise in plasma SP-D levels [62].

KL-6 is a mucin-like glycoprotein expressed on the surface of alveolar type II cells. Damage to pulmonary epithelial cells allows KL6 to diffuse into the pulmonary epithelial lining fluid (ELF). In a study of 32 patients with ARDS, ELF was collected using a bronchoscopic microsampling procedure, showing that KL-6 levels in ELF, but not serum, measured during the early period after the diagnosis were useful for predicting mortality [63].

The receptor for advanced glycation end products (RAGE), a member of the immunoglobulin superfamily that acts as a multi-ligand receptor, has been identified as a marker of alveolar type I cell injury [64]. In a multicenter study of 676 patients with ARDS enrolled in a randomized controlled trial of lower tidal volume ventilation, higher baseline plasma RAGE was associated with increased severity of lung injury and mortality (OR 1.38) [65].

Clara cell protein (CC-16), an inhibitor of phospholipase A2 activity produced by distal bronchiolar epithelial cells, has been shown to have a protective and immunomodulatory role in acute lung injury [66]. In a retrospective study of 22 patients with ventilator-associated pneumonia, levels of CC-16 increased 2 days before ARDS diagnosis [67]. However, Kropski et al. showed that levels of CC-16 in plasma or edema fluid were lower in ARDS than in critically ill controls with cardiogenic pulmonary edema [68]. In a prospective multicenter observational study from Quebec Critical Care Network, a higher initial CC-16 serum level was associated with increased risk of death in 78 adult ARDS patients [69]. Different time points for obtaining the CC-16 and variable severity of lung injury may be potential explanations for these conflicting findings.

3. Inflammatory mediators

Inflammation is fundamental to the pathogenesis of ARDS and a variety of inflammatory biomarkers have been assessed for prognostic utility. Among the cytokines, plasma leukotriene (LT) B4, interleukin (IL)-8, and IL-6 have been recognized as useful prognostic indices in patients with early phase ARDS [70–72]. In addition, serum IL-2 and IL-15 levels had shown to be associated with mortality in patients with ARDS [73].

Decoy receptor (DcR) 3, belonging to the tumor necrosis factor receptor superfamily, is an antiapoptotic soluble receptor considered to play a role in immune modulation [74]. In a study of 88 ARDS patients, high plasma DcR3 levels correlated with development of multiple-organ dysfunction and independently predicted 28-day mortality [75].

T-regulatory cells (Tregs), a subset of CD4+ lymphocytes that express CD25 (IL-2 receptor **α**) as well as the transcription factor Forkhead box protein 3 (Foxp3) on their surface, have been shown to suppress inflammatory, allergic, and autoimmune disorders [76, 77]. D’Alessio et al. identified that CD4 + CD25 + Foxp3+ Tregs serve a fundamental role in mediating resolution of lung injury by modulating innate immune responses [78]. In a prospective study of 47 patients with ARDS, the ratio of Tregs to all CD4+ lymphocytes in bronchoalveolar lavage (BAL) was shown to be an independent prognostic factor for 30-day mortality [79].

C-reactive protein (CRP) has been studied as a marker of systemic inflammation and outcome in a number of diseases. In a prospective study of 177 patients with ARDS, increasing plasma levels of CRP within 48 h of ARDS onset were associated with a better outcome, implying that CRP is not solely a marker of systemic inflammation [80]. From this same study cohort, Rivara et al. further showed that elevated cardiac troponin T (cTnT), a biomarker of cardiac necrosis, was associated with worsened clinical outcomes and certain echocardiographic abnormalities in patients with ARDS [81].

4. Coagulation

Protein C plays an active role in modulating severe systemic inflammatory processes such as sepsis, and trauma via its anticoagulant and anti-inflammatory properties [82]. The first human study regarding protein C in ARDS, which enrolled 45 patients, showed that lower levels of plasma protein C were associated with worse clinical outcomes [83]. Later, in a larger multicenter study of a protective ventilatory strategy in 779 patients with ARDS, lower plasma levels of protein C were an independent risk factor for mortality and adverse clinical outcomes [84]. Based on these findings, Cornet et al. conducted a randomized control trial of intravenous recombinant human activated protein C for 4 days, finding that it did not improve outcomes in patients with ARDS [85]. Similarly, Liu et al. also showed that activated protein C did not improve outcomes from ARDS in a double-blind randomized control trial [86].

Plasminogen activator inhibitor-1 (PAI-1) impairs fibrinolysis by inhibiting tissue plasminogen activator (tPA) and urokinase (uPA). Ware et al. showed that increased plasma levels of PAI-1 are independent risk factors for mortality in 779 patients with ARDS and that the combination of low protein C levels with high PAI-1 levels portended a particularly poor prognosis [84].

Although there have been a number of biological markers investigated for predicting clinical outcomes in ARDS, no single clinical or biological marker is able to predict the outcome accurately. Thus, Ware et al. combined eight biological markers and clinical variables to predict mortality in 549 patients with ARDS from an NIH ARDS Network clinical trial of two levels of positive end-expiratory pressure in ARDS. In that study, a combination of clinical predictors and biomarkers had the best performance for predicting mortality in patients with ARDS compared to clinical predictors or biomarkers alone [13].

### Scoring systems

For describing the severity-of-illness and constructing risk-prediction models in general ICU populations, many scoring systems have been developed, such as the Acute Physiology and Chronic Health Evaluation (APACHE) scores [87–89], the Simplified Acute Physiology Scores [90] and Sequential Organ Failure Assessment (SOFA) scoring [91]. APACHE II score is not only accurate for predicting outcomes in general ICU patients, but also in patients with ARDS [69, 73]. In addition, the APACHE IV score, the RIFLE score (Risk of renal failure, Injury to the kidney, Failure of kidney function, Loss of kidney function, and End-stage renal failure) [92], and GOCA score (Gas exchange, Organ failure, Cause, Associated disease) [93] have all been proved to have prognostic value in predicting mortality in patients with ARDS.

In terms of scoring systems for predicting mortality specifically in ARDS patients, the most commonly used is the Lung Injury Score (LIS), proposed in 1988 by Murray and colleagues [94]. The Lung Injury Score is based on four components: 1) chest radiograph; 2) hypoxemia score; 3) PEEP; and 4) static compliance of respiratory system. Although it was not originally intended as a risk prediction tool, it has been widely used to assess ARDS severity in clinical studies. However, some studies showed that LIS was not independently associated with in-hospital mortality in ARDS patients [8, 11, 13] when ARDS was defined by AECC criteria [2]. By contrast, in a multi-ICU cohort study with 550 ARDS patients diagnosed by Berlin definition, the LIS was associated with increased in-hospital morbidity and mortality [95].

## Conclusions

ARDS is a complex and heterogeneous syndrome. A variety of clinical and biomarker parameters can be used for risk prediction and prognostication. Discovery of prognostic factors for ARDS has enriched our understanding of the pathogenesis of this complex clinical syndrome and has impacted clinical trial design, risk assessment and accurate diagnosis of the syndrome. In this review, we categorized the prognostic factors into four areas including (1) clinical characteristics (2) physiological parameters and oxygenation (3) genetic polymorphisms and biomarkers (4) scoring systems. In terms of clinical characteristics, age and severity of illness scores remain the most robust predictors of outcome while blood type, race and ethnicity may be novel predictors of death in ARDS that require further validation. Pulmonary dead space, ratio of right over left ventricular stroke work, PaO_2_/FiO_2_ ratio, and oxygenation index are proven to be independent predictors of mortality. A number of genetic polymorphisms have been shown to be associated with mortality in ARDS, in genes including angiotensin-converting enzyme, extracellular superoxide dismutase, duffy antigen/receptor for chemokines, and vascular endothelial growth factor. A variety of biomarkers involved in the pathogenesis of endothelial and epithelial injury, inflammatory response and coagulation have been explored. Scoring systems such as APACHE II, GOCA, LIS, and RIFLE score are reliable in predicting mortality in ARDS. Finally, prognostic factors differ for predicting short-term and long-term outcomes, and may differ depending on treatment including low tidal volume, prone position or ECMO intervention. Thus, recent changes in definitions [1] and ventilatory strategies [9] should be taken into account in interpreting older prognostic studies that preceded the low tidal volume era.

Based on the prognostic factors mentioned above, we may better determine subphenotypes of ARDS. Incorporating these distinctions into clinical trial design may allow more effective treatment selection for a particular ARDS phenotype in the future. For example, Calfee et al. showed that ARDS patients with a hyperinflammatory subphenotype identified by latent class analysis of a combination of clinical characteristics and plasma biomarkers had worse clinical outcomes and differential responses to ventilator interventions in two large cohorts [96]. Briel et al. showed that patients with moderate or severe ARDS, but not mild ARDS had an improved hospital survival when using a higher level of PEEP [97]. Guérin et al. found that only in patients with severe ARDS, but not moderate or mild ARDS, early application of prolonged prone-positioning sessions significantly decreased 28-day and 90-day mortality [6]. These studies illustrate the potential for use of prognostic factors including clinical factors such as oxygenation and biological markers for better selecting patients for enrollment in ARDS clinical trials.
